# Impact of uncertainty and ambiguous outcome phrasing on moral decision-making

**DOI:** 10.1371/journal.pone.0233127

**Published:** 2020-05-26

**Authors:** Yiyun Shou, Joel Olney, Michael Smithson, Fei Song

**Affiliations:** 1 Research School of Psychology, The Australian National University, Canberra, Australia; 2 Department of Philosophy, The University of Hong Kong, Pokfulam, Hong Kong; Middlesex University, UNITED KINGDOM

## Abstract

The literature has shown that different types of moral dilemmas elicit discrepant decision patterns. The present research investigated the role of uncertainty in contributing to these decision patterns. Two studies were conducted to examine participants' choices in commonly used dilemmas. Study 1 showed that participants’ perceived outcome probabilities were significantly associated with their moral choices, and that these associations were independent from the dilemma type. Study 2 revealed that participants had significantly less preference for killing the individual when the outcome probabilities were stated using the modal verb ‘will’ than when they were stated using the numerical phrasing of ‘100%’. Our findings illustrate a discord between experimenter and participant in the interpretation of task instructions.

## Introduction

Moral dilemmas that involve trolley-type problems are situations where an agent must decide between killing the minority to save the majority versus allowing the majority to die. Moral dilemmas are thought to produce a conflict between utilitarian normative ethics and deontological normative ethics. Deontology ascribes rightness to actions based on principles and moral duties that are inviolable [1]. Contrarily, the corollary of utilitarian reasoning is the infamous maxim that the ends justify the means, so long as the means produce outcomes that have a net positive gain of goodness across affected individuals [2]. Moral and cognitive psychologists seek to understand the psychological mechanism of the ethical systems to which people subscribe.

Studies of moral preference have consistently found discrepant choice patterns across different variants of moral dilemmas. This is seemingly despite the relative outcomes of the binary choices within these dilemmas being equivalent (e.g., killing one individual versus allowing five people to die). Some dilemmas, such as the *switch* case (see S1 Text for the complete dilemmas), consistently produce high rates of decisions in favor of killing the individual to save the five people at risk. Other dilemmas, such as the *footbridge* variant, consistently produce a higher proportion of judgments in favor of inaction (not saving the five) [2–6].

The inconsistency implies that, instead of a simple dichotomous view that people are either deontological or utilitarian, there may be a certain degree of moral flexibility across contexts [7]. One possible explanation for the discrepancy in choice patterns across moral dilemmas is that moral agents are reasoning under conditions of uncertainty about the outcome probabilities. Kortenkamp and Moore [8] found that participants were more reluctant to indicate that *kill* was appropriate if the outcomes were uncertain than if the outcomes were certain. The authors manipulated uncertainty by describing the outcome as something that “might” happen in the uncertain condition and something that “will” happen in the certain condition. However, the authors of this study assume the word “will” was interpreted as equivalent to “certain” by participants and did not directly test participants’ beliefs about the outcome probabilities. In addition, they only investigated three dilemmas and therefore little is known about differences in this uncertainty effect among different types of moral dilemmas. Epistemic uncertainty can be considered a psychological state in which an individual in a decision scenario does not know if a given alternative/action will necessarily lead to a given outcome [9]. For example, moral agents may not be reasoning as though the action of killing one person will certainly lead to the outcome of saving five people. Instead, subjective probability judgments may underlie the utility functions an individual ascribes to choices in a moral dilemma.

Shou and Song [10] generated two moral dilemmas and asked participants for probability judgments for the four possible outcomes specified in the dilemma:

1. *P* (5*S*|*K*): the probability that five people would survive (5*S*) given killing the individual (*K*) is chosen;
2. *P* (1*D*|*K*): the probability that the individual would die (1*D*) given *K* is chosen;
3. *P* (5D|~*K*): the probability that five people would die (5*D*) given not killing the individual (~*K*) is chosen;
4. *P* (1*S*|~*K*): the probability that the individual would survive (1*S*) given ~*K* is chosen.

Shou and Song [10] showed that participants’ perception of these probabilities was significantly associated with their moral choices. Higher probability judgments of the positive outcome given kill (the five people would survive) or the negative outcome given not kill (the five people would die) were associated with a stronger tendency to choose to kill the individual.

Shou and Song [10] also found that the mean value for participants’ four probability judgments was well below 100%. This, however, contradicts the unstated assumption throughout the literature on moral dilemmas that people reason as though the outcomes are certain.

Reasoning as though outcomes are uncertain in moral dilemmas seemingly involves a rejection of the semantic content of moral dilemmas that stipulate choices ‘*will*’ lead to outcomes. Greene, Cushman, Stewart, Lowenberg, Nystrom, and Cohen [11] asked participants to indicate their real-world expectations regarding the actions leading to outcomes after they had provided moral judgments across a broad set of dilemmas. The researchers found that participants who expected a worse outcome for an action in a scenario endorsed that action less than those whose expectation was more optimistic [11]. The researchers labelled this tendency to compensate for the potentially unrealistic assumptions within the dilemma description with perhaps more subjectively realistic ones as ‘unconscious realism’.

These findings imply that participants may not take the stated premises of outcomes within a dilemma as given, and therefore do not reason under the same premises the experimenter has assumed. It is unclear why participants provided uncertain probability estimates even though the instructions seemed to task participants to reason as though they are certain. One possible explanation for this phenomenon comes from the literature on verbal probability expressions. Verbal expressions of probability can be perceived as more vague and less determinate than numerical probabilities [12]. Although some literature demonstrates the imprecision of verbal probability phrases (e.g., “extremely unlikely” and “extremely likely”) among laypersons’ interpretations and communication, few studies investigate the interpretation and communication of the expression ‘will’. Teigen and Filkuková (2013) suggest that many people hold a probabilistic view of the modal verb ‘will’, as if to describe what one expects rather than what one thinks will definitely transpire.

Furthermore, Teigen and Filkuková [13] found that many people used ‘will’ in statements for a lower bound value. With the use of ‘will’ in the statement, other numbers in the statement were given an ‘at least’ interpretation after modal verbs (e.g., “a battery will last for 1.5 hours”). It seems that some people may use ‘will’ to describe the most likely outcome value even though the outcome likelihood might not be 100%. It is possible that when people read a ‘will’ statement as recipients, they may translate the statement to probabilities lower than 100% or assume an outcome value different from the value in the statement in certain situations. Therefore, to tell participants that particular actions *will* lead to particular outcomes in moral dilemmas, as has been the practice within the literature hitherto, is to leave room for them to reason under conditions of uncertainty. Consequently, participants may infer the likelihood of the outcomes based on subjective probabilities.

In addition, it is unclear if there are individual differences in terms of being influenced by subjective perceptions of the four probability estimates. For example, moral judgments are considered to be associated with uncertainty [10,14], rational and intuitive thinking style [1], and endorsement of consequentialist ethics [15]. Uncertainty-averse participants might be more sensitive to the perceived uncertainty (probabilities) of outcomes, thus a higher risk-averse tendency can result in stronger associations between perceived probabilities of outcomes and moral decisions. Furthermore, individuals who prefer a rational thinking style may be more influenced by outcome probabilities in their decision making, thus strengthening the associations between perceived probabilities of outcomes and moral decisions. Finally, individuals who have a stronger endorsement of consequentialist ethics may be more influenced by outcome probabilities as a result of being sensitive to the change in outcomes. These individuals may be more influenced by these probabilities when making moral decisions.

Without controlling for the probabilities of outcomes in different variants of dilemmas, any conclusions regarding a particular feature in a dilemma influencing moral choices can be confounded. One dilemma feature commonly discussed in the literature is personal force (e.g., using one’s own muscles to harm is up-close, personal, and therefore affect-laden; [10]). The early classification of dilemmas, being either personal or impersonal, stems from early work on the dual process model by Greene et al. [5] which argued that a prepotent and aversive emotional response brought about by particular dilemmas interrupts the ability for rational reasoning and results in a lower likelihood of choosing to kill in a dilemma [5]. In Greene and colleagues’ [11] more recent work, the authors classify those dilemmas that bring about this response as dilemmas that involve “personal force”, a combination of the directness and intention of the harm brought about (such as in the *footbridge* dilemma). The authors argue that this type of action may trigger higher aversive emotional responses, thus resulting in a higher preference for not killing. Shou and Song [10] found that for the hostage dilemma that involved personal force, participants ascribed higher probability values to the negative outcome given kill (the individual would die) and the positive outcome given not kill (the individual would survive) than they did for the car dilemma that did not involve personal force. Similarly, for the car dilemma participants ascribed higher probability values to the positive outcome given kill (the five people would survive) and the negative outcome given not kill (the five people would die). Thus, the distinction in choice preference between impersonal and personal dilemmas can be confounded with uncertainty about the outcomes in moral dilemmas.

## The present study

The main purpose of this study was to explore factors that can contribute to subjective probability judgments. We explore this from two approaches. First, we investigate individual difference covariates, including attitude toward risk, general endorsement of consequentialist ethics, and general rational-experiential thinking style. Second, we explore the explanation of vagueness in verbal probability phrases and examine whether participants interpret the word ‘will’ as equivalent to 100%.

In addition, the previous study utilized two dilemmas generated by the authors [10] and therefore it is unclear if the results can be generalized to other commonly used moral dilemmas. The present study included a range of commonly used dilemmas and examined the generalizability of the previous findings. We also investigated how probability judgments vary across dilemmas, and whether the differences are associated with a feature such as the personal/impersonal nature of the dilemmas. More specifically, we aim to verify that:

1. Participants reason as though the outcomes brought about by actions are not certain in moral dilemma judgment tasks;
2. Participants’ probability judgments vary across different moral dilemmas, specifically between personal and impersonal dilemmas. The probability judgments of the positive (negative) outcomes for the option of kill will be higher (lower) in the impersonal dilemmas than they are in the personal dilemmas. Similarly, the probability judgments of the positive (negative) outcomes for the option of not kill will be higher (lower) in the personal dilemmas than they are in the impersonal dilemmas;
3. Participants’ moral choices will be significantly associated with their perceived likelihood of outcomes in the dilemma; the probability judgments of the positive outcome of an action (kill, not kill) will be positively associated with the endorsement of that action. On the other hand, the probability judgments of the negative outcome of an action will be negatively associated with the endorsement of that action. Finally, that these associations will not be significantly different between personal and impersonal types of dilemmas.

Two studies were conducted. Study 1 investigates how participants interpret the likelihood of outcomes in moral dilemmas, and how their subjective perception of outcome probabilities influences their moral choices. In addition, Study 1 investigates whether probability judgments can account for the variability in choice preference between personal and impersonal moral dilemmas. Study 2 investigates the imprecision of verbal descriptions of probabilistic information in moral dilemmas.

## Study 1

Study 1 emulated the method of Shou and Song [10]. Participants provided moral judgments and likelihood estimations regarding the four aforementioned potential outcomes. The study employed a within-subjects design (2 x 9 dilemma type conditions), where each participant completed a moral choice task and probability judgments for 18 dilemmas. The 18 dilemmas were drawn from previous studies, and nine were traditionally classified as personal dilemmas and the other nine as impersonal dilemmas (from [11]).

The first purpose of Study 1 was to examine the perceived outcome probabilities in the 18 moral dilemmas. We expected that (H1a) not all participants would perceive the outcomes as certain. In addition, we expected that (H1b) the probabilities would vary across dilemmas; specifically, probabilities would differ significantly between personal and impersonal types of dilemmas. Based on previous findings, we expected that the probability judgments of the positive outcomes given kill, *P*(5*S|K*), and the probability judgments of the negative outcomes given not kill, *P*(5*D|~K*) will be higher in the impersonal dilemmas than they are in the personal dilemmas; while the probability judgments of the negative outcomes given kill, *P*(1*D|K*), and the probability judgments of the positive outcomes given not kill, *P*(1*S|~K*), will be higher in the personal dilemmas than they are in the impersonal dilemmas.

The second aim of Study 1 was to examine the associations between the perceived outcome probabilities and participants’ moral decisions. We hypothesized that (H2) preferred choice in the set of 18 moral dilemmas is associated with participants’ perceived probabilities regarding positive and negative outcomes. Specifically, higher perceptions of the likelihood of positive outcomes and lower perceptions of the likelihood of negative outcomes would be associated with stronger endorsement of that choice. We also investigated if the associations differ between the two types of dilemma. It is hypothesized that (H3) the associations between subjective probabilities and moral choice are not moderated by the type of dilemma (personal, impersonal).

Furthermore, we included covariate scales that measure attitudes toward uncertainty in the ethical domain, the preference for rational and intuitive cognitive styles, and subscription to consequentialist ethics. We examined if these individual difference traits could moderate the impact of probability estimates on choice behaviours. We expected that associations between perceived probabilities of the outcomes and moral decisions would be stronger among participants who have a higher risk-averse tendency, who have a stronger endorsement of consequentialist ethics, and who prefer a rational thinking style.

## Method

### Participants

A total of 106 participants (56% female) were recruited via CrowdFlower, an online crowdsourcing platform. Participants were aged between 18 and 78, with a mean of 37 (*SD* = 11.66). Participants were paid US$1 for participation in the study. Twenty-two cases were excluded due to failure to correctly answer catch questions. This left a total of 84participants (63% female, *M* = 38.21, *SD* = 11.95, age range = 19–64). We had a repeated design in which each participant provided 18 data points (see Materials for more details). Based on a 20:1 data points to parameter ratio rule, a minimum of 45 participants would be required for a reliable estimation of a fixed effect model that include all possible factors and interactions in Study 1 (approximately 20 parameters). Thus, 84 participants were sufficient for the analysis requirement. Approximately 65.5% of the participants had completed at least a tertiary degree. The ethical aspects of this research have been approved by the Australian National University Human Research Ethics Committee (Protocol 2017/349).

### Materials

The survey was constructed using Qualtrics^TM^ and consisted of three main components: general demographic information, moral judgment tasks, and the covariate scales. The demographic page recorded gender, age, level of education, and religious affiliation.

#### Moral judgment tasks

The nine personal and nine impersonal dilemmas were taken from both Greene et al. [2], and Christensen et al. [3]. Each moral dilemma was standardized so as to present the problem as a five-for-one person exchange should the kill option be chosen.

In each dilemma, participants read the dilemma scenario and completed a moral choice question followed by four probability judgment questions. The choice question read *‘which do you think is morally better*?’ followed by two options: a description of the kill option and a description of the not kill option. In this study, moral preference was referred to explicitly, as opposed to appropriateness or permissibility, in order to draw attention to moral considerations and away from legal, conventional, or other considerations that may not necessarily entail a moral flavour [8,16]. Previous research has demonstrated that how the judgment question was worded can influence people’s choice endorsement. For example, phrases such as “permissibility” invoke more legal and convention considerations in the task, while asking whether something “should” be done may emphasize the utilitarian nature of the action and thus lead to harsher moral judgments than other phrases [17].

The decision alternatives were worded in such a way as to minimize either positive or negative framing effects. Rather than using loaded words, such as kill or save (see [18]), the options were presented as neutrally as possible, such as ‘*comply with the tribal leader's request*’ or ‘*do not comply with the tribal leader's request*’. Where neutral wording proved more difficult, some decision options were worded in such a way as to reiterate the ‘good’ consequences of the ‘bad’ action (as suggested by [19]), such as ‘*throw the injured person overboard to stop the boat from sinking*’.

The four probability judgment questions asked for participants’ subjective beliefs regarding the probability of the positive outcome given kill *P*(5*S|K*), the probability of the negative outcome given kill *P*(1*D|K*), the probability of the positive outcome given not kill *P*(1*S|~K*), and the probability of the negative outcome given not kill *P*(5*D|~K*). The order of the 18 dilemmas in the main task was randomized.

#### Post-hoc justification check

Ideally, the probability judgments should not be influenced by the decisions that participants had made. However, it is possible that the process of choosing which moral judgment participants preferred before assigning probability judgments could affect the probability judgments in the form of post-hoc justification [20]. This post-hoc justification would invalidate the subjective probabilities obtained, as the probability judgments would no longer represent participants’ natural beliefs about how likely they believed outcomes were.

In order to address this issue, four moral dilemmas (2 personal, 2 impersonal; *footbridge*, *burning*, *car*, and *fumes–*see S1 Text) were selected to experimentally test whether post-hoc justification of choice behavior was significantly impacting participants’ subjective probabilities. The test involved three within-subject conditions. Participants’ responses to the four dilemmas in the primary moral judgment task were considered the normal condition, where each dilemma incorporated the standard binary decision alternatives: kill or not kill. The four dilemmas were repeated both in a *preferred* condition and a *not preferred* condition, each of which had three decision alternatives in total, which included the standard binary decision alternatives.

The *preferred* condition contained a third option that was designed so as to be highly preferred to the standard decision alternatives (e.g., ‘activate a ventilation lock-down that stops the spread of the fumes to any of the rooms’). In the *preferred* condition, where the participants should have favored neither of the traditional options (*K*, *~K*) because of a more desirable option, the subjective probabilities acquired regarding *K* and *~K* for these dilemmas were assumed to have not been influenced by post-hoc justification of the moral judgment they had just given.

However, because the inclusion of a subset of dilemmas with a preferred choice partitions the suggested state space from two (*K*, *~K*) to three (*K*, *~K*, preferred; see [21]), the *not preferred* condition was set up in order to measure any probability differences due to changes in that state space. The *not preferred* condition also contained three options, of which the third option was designed to be highly undesirable and not preferred by participants in comparison to the traditional choices (e.g., ‘activate the ventilation fan causing the toxic fumes to spread throughout the entire hospital’).

If the subjective probabilities from the *normal* condition were not significantly different from the probabilities in the preferred and not preferred conditions, it could be assumed that post-hoc justification and the partition of the suggested state space were not substantially influencing the data. This would suggest that probabilities in the *normal* condition could be considered participants’ natural beliefs.

#### Covariate scales

Three covariates were included in the study. First, the ethical subcomponent of the Domain-Specific-Risk-Taking (DOSPERT) scale [22] is an eight-item scale and measures attitudes towards ethical risks (risk-averse to risk-seeking). Each item specifies an unethical behavior (e.g., cheating on an exam) and is rated on a 5-point Likert scale from 1 (very unlikely) to 5 (very likely) in engaging in that behavior (Cronbach’s alpha = .80). Second, the Consequentialist-Thinking Scale [15] is a 13-item scale measuring the construct of trait consequentialism, or moral cognitive style (Cronbach’s alpha = .89). Each item names a behavior (e.g., Torture) and asks participants to rate their position as 1 (Never morally permissible), 2 (morally permissible if it leads to more good than bad consequences), or 3 (morally obligatory if it produces more good than bad consequences). Lastly, the Rational-Experiential Inventory [23] is a 10-item scale that measures two subscales consisting of 5 items each: the preference for logical-rational information processing (Cronbach’s alpha = .79) and the preference for intuitive information processing (Cronbach’s alpha = .88). Each item has a statement about thinking style (e.g., ‘I trust my initial feelings about people.’, and ‘I prefer complex problems to simple problems.’) and is rated on a 5-point Likert scale from 1 (strongly disagree) to 5 (strongly agree).

#### Procedure

Participants were redirected from CrowdFlower via link to Qualtrics^TM^ in order to complete the survey. Participants were provided with the online information sheet first and proceeded if they agreed to participate. Participants provided their consent to participate in the research by completing and submitting the survey. Participants completed the demographics questionnaire, the main moral judgment tasks, and covariance scales, in that order. Participants were then debriefed, thanked, and given a code that would allow them to receive the remuneration.

## Results

### Probability judgments across dilemmas

Table 1 shows the descriptive statistics of the probability ratings. As we expected, the mean probability ratings for the four possible outcomes for each dilemma were below 100% despite the modal verb ‘will’ being used within the dilemmas. Only a small percentage of cases indicated that they believed ‘will’ translated to 100% certainty for *P* (5*S|K*) (13.6%), *P* (1*S|~K*) (16.1%), and *P* (5*D|~K*) (14.4%), whereas a larger proportion indicated that *P* (1*D|K*) was 100% certain (37.8%).

**Table 1:** Descriptive statistics for individual moral dilemmas in study 1.

	%Choosing *K* (*n*)	Proportion of rating 100%				Mean probability rating			
Dilemma		*1*	*2*	*3*	*4*	*1*	*2*	*3*	*4*
Impersonal Dilemmas									
Switch	55(46)	41.7	34.5	40.5	21.4	83.6	80.3	78.2	77.1
Fumes	54(45)	25.0	34.5	32.1	22.6	82.3	85.3	79.9	78.1
Shipyard	83(70)	32.1	14.3	28.6	19.0	82.1	70.9	79.9	75.6
Car	61(51)	27.4	23.8	41.7	15.5	82.3	82.1	85.8	75.2
Floods	56(47)	17.9	39.3	2.4	13.1	78.5	86.3	44.6	75.1
Miners	57(48)	10.7	38.1	6.0	13.1	76.3	84.8	53.6	72.9
Scaffolding	50(42)	10.7	33.3	4.8	9.5	73.9	84.6	56.0	71.5
Bikers	51(43)	3.6	19.0	8.3	8.3	72.3	76.2	66.6	63.5
Sharks	39(33)	2.4	40.5	2.4	6.0	71.2	84.4	61.3	65.7
Personal Dilemmas									
Burning	32(27)	6.0	32.1	1.2	9.5	76.2	85.0	68.6	70.8
Lifeboat	51(43)	8.3	35.7	1.2	8.3	72.8	85.0	51.7	69.9
Submarine	39(33)	8.3	47.6	2.4	9.5	72.9	84.7	51.6	70.2
Plane crash	24(20)	6.0	63.1	4.8	4.8	70.2	89.4	57.1	63.3
Transplant	23(19)	10.7	54.8	48.8	16.7	70.2	89.5	84.2	74.6
Footbridge	24(20)	10.7	45.2	53.6	17.9	68.5	86.4	86.8	74.5
Crying baby	62(52)	7.1	23.8	3.6	22.6	67.5	74.6	42.8	81.1
Sacrifice	25(21)	8.3	52.4	3.6	17.9	61.7	88.5	41.4	74.1
Safari	28(24)	8.3	47.6	3.6	23.8	59.3	85.6	40.8	78.1

1 = P (5S|K); 2 = P (1D|K);3 = P (1S|~K), 4 = P (5D|~K).

**Fig 1:**
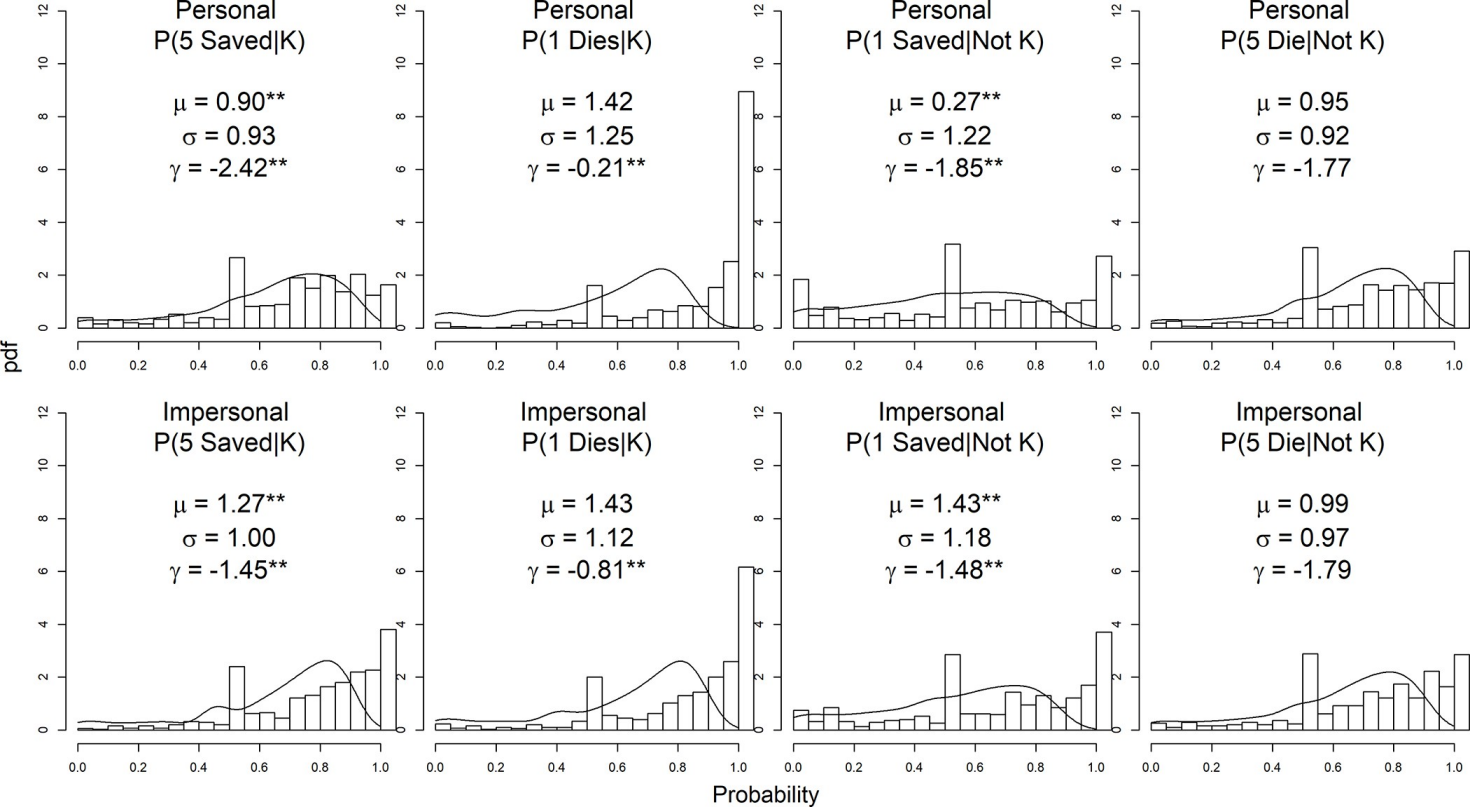
Histogram of the probability judgments across different probability types and dilemma types, and fitting results of the one-inflated T2-logistic models. The solid lines are fitted T2-logistic distribution curves. **p < .001.

Inspecting the probability distributions (see Fig 1) demonstrates that the four probabilities across the two types of dilemmas are all non-normally distributed, being highly skewed and with a consistent mode at 0.5. We fit the probability judgments using one-inflated T2-logistic distributions. The T2-logistic distribution is a member of the CDF-quantile family distributions [24] for modelling variables on (0, 1) interval. The CDF-quantile family captures a wide variety of shapes and the T2-logistic distribution is able to account for skewed and trimodal shaped distributions and achieved best average fit values across different probability judgments (for more details see [24]). The one-inflated T2-logistic GLMs have three parameters: mu, sigma and gamma, which correspond to the median, dispersion and the likelihood of being 1, respectively. A one-inflated T2-logistic GLM has three submodels, each of which links a parameter with a linear combination of predictors via a certain link function. Thus, we can test if there are significant differences in means and/or dispersions (variability) across samples.

Table 2 shows parameter estimation results for each probability judgment fit by the T2-logistic distribution, ordered by the value of gamma. A greater positive value for gamma indicates a higher likelihood that the participants would rate 1 for that probability judgment. A greater value for mu indicates a higher median in probability judgments excluding a 1 rating. Finally, a greater value for sigma indicates a greater dispersion, or variability. As seen from the table, both gamma and mu vary across probability types and dilemma types, indicating large variation in probability judgments. The likelihood of choosing a probability of 1 for personal dilemma outcome judgments ranged from the highest in the rank (45%) to the lowest in the rank (8%).

**Table 2:** Estimated results for the probability estimated ranked from the highest estimated mean to the lowest estimated mean.

Dilemma	Probability	μ	σ	γ	*P*(1)	Log likelihood
personal	*P*(1*D|K*)	1.42	1.25	-0.21	0.45	-392.08
impersonal	*P*(1*D|K*)	1.43	1.12	-0.81	0.31	-303.49
impersonal	*P*(5*S|K*)	1.27	1.00	-1.45	0.19	-189.49
impersonal	*P*(5*D|~K*)	0.99	0.97	-1.79	0.14	-183.76
personal	*P*(5*D|~K*)	0.95	0.92	-1.77	0.15	-176.10
personal	*P*(1*S|~K*)	0.90	0.93	-2.42	0.08	-93.35
impersonal	*P*(1*S|~K*)	0.62	1.18	-1.48	0.19	-324.38
personal	*P*(5*S|K*)	0.27	1.22	-1.85	0.14	-279.32

*P* (1) is the percentage of responses rating at 100% for a type probability rating.

For each probability type, we also tested the differences in the three parameters between the personal and impersonal dilemmas. The significant test results are starred in Fig 1. As illustrated, for the outcomes of killing, participants perceive the likelihood of the positive outcomes (5 people saved) as substantially higher in impersonal dilemmas than in personal dilemmas, while the likelihood of the negative outcomes (the individual dies) as substantially higher in personal dilemmas than in impersonal dilemmas.

### Associations between moral choice and probabilistic reasoning

Table 1 also presents the proportion of participants choosing *K* across different dilemmas. Consistent with previous studies [2–4], choice behavior differed substantially across dilemma type (DT), with impersonal dilemmas eliciting higher endorsement of *K* choice behavior than personal dilemmas.

Mixed-effects binary logistic regression was used to test the association between the perceived likelihood of outcomes and moral choice across the 18 dilemmas, and whether this association was moderated by the dilemma type. Analyses were performed using the “lme4” package [25] in R (version 3.3.0). The significance of the terms was tested with a chi-square test between a model with the term and a model without the term.

An initial null model was constructed to predict the endorsement of the moral choice *K* (kill) over *~K* (not kill) from the four probability judgments and DT (Log likelihood = -754.33, AIC = 1523; DT was effects coded: personal = -1, impersonal = 1). Entering the interaction effects between the four probability judgments and DT did not significantly improve the model fit (χ^2^ = 7.75, df = 4, *p* = .101). The four covariate scales (standardized) and their interactions with the four probability judgments or DT were also examined for their contribution to the model. Only three interactions were found to be significant (see Table 3). Table 3 shows the estimation of the results for the final model (Log likelihood = -733.3, AIC = 1495).

**Table 3:** Mixed-effects binary logistic regression predicting endorsing the kill option.

Parameter	*b*	*SE*	*p*	exp(b)
Intercept	-0.33	0.16	.038	0.72
*P*(5*S|K*)	0.73	0.11	< .001	2.07
*P*(1*D|K*)	-0.65	0.10	< .001	0.52
*P*(1*S|~K*)	-0.43	0.08	< .001	0.65
*P*(5*D|~K*)	1.00	0.11	< .001	2.53
DT	0.63	0.08	< .001	1.88
DOSPERT	0.27	0.20	.163	1.31
Rational Thinking	-0.05	0.16	.779	0.96
Consequentialist	0.26	0.19	.181	1.30
*P*(1D*|K*) x DOSPERT	0.28	0.10	.003	1.33
*P*(1S*|~K*) x Rational Thinking	-0.20	0.08	.010	1.22
*P*(5*D|~K*) x Consequentialist	0.27	0.11	.019	1.31
DT x DOSPERT	-0.21	0.07	.001	0.81
Random intercept	1.274			

The dependent variable is the moral choice on endorsing the kill option versus not kill option, where the not kill option was set as the base level. DT = 1 refers to impersonal dilemmas, and = -1 refers to personal dilemmas. DOSPERT (Domain-Specific-Risk-Taking-Ethical scale), Rational Thinking (Rational-Experiential Inventory), Consequentialist (Consequentialist-Thinking Scale) were standardized scores.

The hypothesis that participants’ preferred choice option across the set of 18 moral dilemmas would be significantly associated with their perceived outcome probabilities was supported. *P* (5*D|~K*) and *P* 5*S|K*) were positively associated with the likelihood of endorsing *K*, while *P* (1*D|K*) and *P* (1*S|~K*) were negatively associated with the likelihood of endorsing *K*. One standard deviation increase in the estimations of *P* (5*D|~K*) and *P* (5*S|K*) resulted in 2.53 and 2.07 times the odds of endorsing K, respectively. Similarly, one standard deviation decrease in the estimations of *P* (1*D|K*) and *P* (1*S|~K*) resulted in 1.92 and 1.54 times the odds of endorsing *K*, respectively.

In addition, we found that attitudes towards unethical behavior (DOSPERT) significantly moderated the effect of *P* (1*D|*K). For individuals who were at the average level of risk-seeking (at mean score of the DOSPERT), higher *P* (1*D|K*) was associated with lower endorsement of the utilitarian choices (*b* = -0.65). The negative relationship was amplified when risk-seeking decreased (or risk aversion increased). For individuals who scored 1 standard deviation below the mean of the DOSPERT scores, the association strength between *P*(1*D|K*) and likelihood of choosing kill increased to *b* = -0.93. Thus, higher risk-averse attitudes strengthened the negative association between *P* (1*D|K*) and the endorsement of *K*.

We also found a significant interaction between the personal endorsement of consequentialist ethics and *P* (5*D|~K*). Higher endorsement of consequentialist ethics strengthened the association between *P*(5*D|~K*) and the endorsement of *K*. Individuals who have a higher endorsement of consequentialist ethics were more inclined to choose *K* when they perceived a higher likelihood of the negative consequences of not killing (five people would die). Finally, a significant interaction between rational thinking style and *P* (1*S|~K*) was found. Individuals who had a higher score on rational thinking style were more sensitive to the likelihood of the positive consequences of not killing in making decisions.

### Probability judgments and post-hoc justification check

CDF-quantile regression using the T2-logistic distribution was applied to examine if the four probability judgments differed significantly as a result of condition (*normal*, *preferred*, *not preferred*) in a subset of four dilemmas (*car*, *fumes*, *burning*, *footbridge*). The four probabilities were tested separately as the dependent variable in the model, and dilemma type and condition (*normal*, *preferred*, *not preferred*) were used as the independent variables.

For each probability judgment, an initial model was constructed including dilemma type in both location and dispersion submodels. A second model was then constructed by adding condition to both submodels. A third model had the interaction term between dilemma type and condition included in both submodels. Likelihood ratio tests were used to examine if the model fit had been substantially improved with the inclusion of condition and the interaction term.

Results indicate that condition did not have significant contributions to the model fit for all four probabilities, χ^2^ (df = 4) ≤ 7.05, *p*s ≥ .133. This suggests that the probability judgments were not significantly different between the different conditions. In addition, the third model was not significantly better than the second model for all four probability judgments, χ^2^ ≤ 8.53, *p*s ≥.748.

### Study 1 discussion

First, as we expected in H1a, participants’ probability estimates for the outcomes in the moral dilemmas were mostly under 100% despite being instructed that outcomes ‘will’ happen. In addition, and in accordance with H1b, participants’ perceived outcome probabilities varied across outcome types and dilemma types. Notably, participants were more likely to rate 100%, and had higher probability judgments, for *P* (5*S|K*) in impersonal dilemmas than they did in personal dilemmas. This indicates that participants generally had greater certainty that the five people would survive if they decided to kill the individual in impersonal dilemmas compared to personal dilemmas. On the other hand, participants had a greater tendency to rate 100% for *P* (1*D|K*) in the personal dilemmas than they did for the impersonal dilemmas, indicating they were more sure that the individual would die if *K* was chosen in the personal dilemmas.

The second purpose of Study 1 was to examine whether subjective probability beliefs concerning the outcomes of choice options could be linked to the choice behavior in moral dilemmas. The results demonstrated that participants’ subjective probability judgments significantly correlated with their moral choice. The results imitate those of Shou and Song [10], and the authors contended that consequentialist reasoning was taking place even though decisions to not kill had been made. The results show that probabilistic reasoning can be closely related to choice behavior across a broad set of moral dilemmas for decisions to both kill and not kill. Furthermore, this association between choice behavior and probability judgments was not moderated by dilemma type (H3).

In addition, we found risk attitudes (DOSPERT) significantly moderated the association between *P* (1*D|K*) and the endorsement of *K*. Individuals who were more risk averse were more sensitive to their perceived probability of the loss given *K*. Participants with a higher risk-averse tendency were more reluctant to choose *K* when they perceived a higher likelihood that the one person would die. On the other hand, people who have stronger risk taking tendency are more tolerant to the uncertainty of loss. The association between *P* (1*D|K*) and the endorsement of *K* was attenuated for participants who scored higher on the DOSPERT.

Another covariate effect found was the significant interaction between the personal endorsement of consequentialist ethics and *P* (5*D|~K*). Individuals who have a higher endorsement of consequentialist ethics were more likely to choose *K* when they perceived a higher likelihood of the negative consequence of not killing. It reflects that strong consequentialists emphasize on minimizing harm/loss and can be more loss averse that those who do not strongly endorse consequentialist ethics. Finally, we found a significant interaction between logical rational information processing and *P* (1*S|~K*). Individuals reporting a greater preference for rational thinking had greater consideration of the likelihood that the individual would survive given not killing when making the judgement.

One important finding from Study 1 warranted further investigation. It was found that participants, on average, provided probability judgments that were below 100% despite being instructed within the content of the dilemma to reason as though outcomes ‘will’ happen. This finding represents a potentially serious discrepancy between the assumptions taken for granted by an experimenter in using the word ‘will’ and participant interpretation of ‘will’. However, it remains unclear if choice behavior tendencies can be changed by manipulating the form of the probabilistic information to be perceived as more certain, hence reinforcing the initial finding that ‘will’ is treated as uncertain (i.e., <100%). In addition, the fact that participant probability ratings were related to their moral judgments suggests that altering the specified outcome probabilities, for example, to 100%, could change participants’ behavioral tendencies. This issue is explored further in Study 2.

## Study 2: Communicating certainty verbally vs numerically

Study 1 suggested that the use of the modal verb ‘will’ within moral dilemmas did not necessarily mean participants were reasoning as though outcomes were certain. Verbal phrases indicating probability can be perceived as vague and are liable to be construed with a degree of variability between individuals [26], whereas numerical probabilities are perceived as more accurate, stable, and reliable [12].

A previous study conducted by Kortenkamp and Moore [8] replaced the modal verb ‘will’, as in ‘such an event *will* happen’, with ‘might’, as in ‘such an event *might* happen’, so as to reflect a lower level of certainty. It was found that participants’ judgments of both the appropriateness and morality of the utilitarian action were significantly lower in the ‘might’ condition as opposed to the ‘will’ condition. While the findings of Kortenkamp and Moore [8] suggest that participants in general perceive ‘will’ as more certain than ‘might’, there was no clear evidence that all participants perceived ‘will’ as 100% certain. Study 2 was carried out to test if participants perceived that the framing of the word ‘will’ was different from (i.e., less certain than) ‘100%’ when interpreting the outcomes.

Study 2 had two conditions: a numerically certain condition and a verbally certain condition. The numerically certain condition presented the four possible outcomes as ‘100%’ certain, while the verbal condition used the modal verb ‘will’ to infer 100% certainty. If the described outcome probabilities were strongly associated with participants’ moral choices, and ‘will’ is interpreted as reflecting a lower probability than ‘100%’, it is hypothesized that outcomes that are presented as verbally certain (‘will’) elicit significantly lower endorsement of K than outcomes presented as numerically certain (‘100%’).

### Method

A total of 300 participants were recruited via Prolific. Sixteen were excluded as they failed the validation question and a further 12 participants were excluded due to having poor or moderate level of English language ability. This left a total of 272 participants for analysis. All participants were fluent in English (70.8% spoke English as a first language). The sample displayed a reasonable gender balance (47.8% female) and age distribution (*M =* 37.17, *SD =* 19.41, age range = 18–67). Participants were offered GBP £ 0.8 remuneration for participation in the study. Participants were redirected from Prolific via link to Qualtrics. Participants were provided with the online information sheet first and proceeded if they agreed to participate. Participants provided their consent to participate in the research by completing and submitting the survey.

A subset of moral dilemmas including four personal (*footbridge*, *safari*, *burning*, *lifeboat*) and four impersonal (*switch*, *miners*, *bikers*, *floods*) dilemmas was selected for the moral judgment task in this study. There were two between-subjects conditions. The verbal, or ‘will’, condition (n = 138) described the outcomes using ‘will’. For example, ‘Divert the train (the individual will die) so that it avoids the five workers (the five workers will survive)’. The numerical condition (*n* = 134) presented the four possible outcomes with numerical probabilities. For example, ‘Divert the train (100% chance of one death) so that it avoids the five workers (100% chance five workers survive)’. Participants read the description of each dilemma and chose the option they perceived as morally better. The order of the eight dilemmas was randomized. In addition, we included the ethical subcomponent of the Domain-Specific-Risk-Taking (DOSPERT) scale to examine if the perception of the verbal term ‘will’ was associated with participants’ risk taking tendency.

### Results

Table 4 presents the endorsement of K among participants across the two conditions. As shown in Table 4, the percentage of participant endorsement of K is higher in the ‘100%’ condition than in the ‘will’ condition.

**Table 4:** Choice behavior when outcome probability information is presented linguistically and numerically.

	Percentage of Endorsing the Kill Option		
Dilemma	‘will’ condition	‘100%’ condition	Odds-ratios
Bikers	70.29	80.60	1.756
Floods	78.99	89.55	2.279
Miners	81.88	87.31	1.523
Switch	78.26	82.09	1.273
Burning	52.90	67.16	1.821
Footbridge	35.50	43.28	1.386
Lifeboat	71.02	81.34	1.779

Mixed-effects binary logistic regression was used to test that the presentation of outcome information would be significantly associated with moral choice. Effects coding was used to generate a variable that indicated which participants were from which condition (numerical condition = -1, verbal condition = 1) and a variable to indicate DT (impersonal = 1, personal = -1). The endorsement of K was used as the DV in the model.

Results demonstrated that the verbal versus numerical condition had a significant main effect on participants’ moral choices (χ^2^ = 9.42, df = 1, *p* = .002). Presenting the outcome information as numerically certain resulted in 4.3 (*b* = 0.77, *p* = .003) times greater odds of endorsing K than the verbal condition.There was also a significant main effect of DT (χ^2^ = 175.6, df = 1, *p* < .001). Participants had 4.44 (*b* = 0.79, *p* < .001) times greater odds of endorsing K in the impersonal dilemmas than in the personal dilemmas. There was no significant interaction between the verbal/numerical condition and the dilemma type DT (χ^2^ = 0.87, df = 1, *p* = .350). Furthermore, DOSPERT did not have a significant main effect or interaction effect with other variables (χ^2^ < 1).

### Study 2 discussion

As hypothesized, significant differences were found in choice behavior depending on the presentation of the probabilistic information. That the endorsement of sacrificing was significantly lower when the probabilistic information was presented verbally indicates that participants in standard moral dilemma tasks do not treat the modal verb ‘will’ as equivalent to 100% certainty. Consequently, many participants are reasoning about action-outcome pairings under perceived uncertainty, and therefore infer their own subjective probabilities concerning the outcomes.

It is also apparent that the endorsement of ‘*K*’ but not ‘~*K*’ increased for all dilemmas despite the probability of the positive outcome for ‘~*K*’ also being specified as 100%. This can be due to the fact that the naturally interpreted probability *P* (1*S*|~*K*) was on average higher than *P* (5*S*|*K*) (see Table 2 and Fig 1). This means that, in the will condition, *P* (5*S*|*K*) could result in a lower mental interpretation than *P* (1*S*|~*K*). When both probabilities were increased to 100% in the numeric condition, the shift in probability estimate is larger for *P* (5*S*|*K*) than for *P* (1*S*|~*K*). When the shift in probability estimates is translated to a shift in the expected values, the action K received a larger increase in expected values than ~*K* did. Consequently, the proportion of the participants choosing *K* increased in the numeric condition.

In addition, it can also be noticed that the proportions of participants choosing *K* in the ‘will’ condition were greater than what were observed in Study 1 for the same sets of the dilemmas. It is possible that the dilemmas in Study 2 had ‘surely’ (i.e., ‘will surely’) in the description which strengthened the statement of the outcomes. Participants might perceive ‘will surely’ as closer to 100% in comparison to ‘will’.

Nevertheless, the findings suggest that many people may equate ‘will’ with only low or moderate numerical probabilities. In combination with Study 1, these findings suggest that the assumption that people reason about classic moral dilemmas as if the outcomes are entirely determined by their choices is unjustified. Instead, many people are reasoning as though chance, or uncertainty, is also involved.

## General discussion

The present paper extended the investigation by Shou and Song [9] into the role of uncertainty in moral dilemmas, and how uncertainty may influence moral judgments. The results replicated previous findings and demonstrated that most participants did not reason as though the outcomes brought about by actions are 100% certain across different moral dilemmas. In addition, the perceived likelihood of the positive outcomes of an option was positively associated with the endorsement of that option, while the perceived likelihood of the negative outcomes was negatively associated with the endorsement of that option.

When exploring the factors contributing to variation in probability judgments, we found that not all participants perceive ‘will’ as 100% certain in the statements forecasting outcomes. They showed significantly less preference for sacrificing when the outcome probabilities were stated using ‘will’ than when they were stated using ‘100%’. ‘Will’ can be perceived as an ambiguous term as it has multiple uses/meanings in commonplace discourse, including ‘expressing inevitable events’ (where the likelihood can be 100%), ‘expressing the future tense’ (where the likelihood can vary), or ‘expressing probability or expectation about something in the present.’ [27].

Participants’ probability judgments varied greatly across different variants of moral dilemmas. It has long been established that people differ considerably in their numerical translations of probability phrases and related quantifiers [28]. People receiving verbal probability expressions (e.g., in forecasts of events) also interpret them as less extreme and less exact than usually is intended by those conveying these messages [29,30], which is what has been observed in Studies 1 and 2 for interpretations of ‘will’. Study 2 showed that participants were more likely to make consequentialist choices when a specified high numerical probability was provided instead of a verbal expression such as the word 'will'. The message for researchers in this domain is clear: if they want to control the probabilities that participants have in mind for a scenario, they should either avoid using verbal probability expressions or, if unavoidable, accompany them with a numerical specification.

The ambiguity of ‘will’ permits the interference of participants’ prior beliefs or experiences relating to the dilemma scenarios when making judgements in hypothetical moral dilemma scenarios. There is an extensive literature on the influence of prior beliefs and experiences on reasoning, such as causal reasoning, syllogistic reasoning, inductive/deductive reasoning, and conditional reasoning [31–33]. Prior beliefs and knowledge can assist in task performance when the beliefs do not conflict with the task [34–36]. Conversely, prior beliefs may produce ‘bias’ in the sense that participants tend to follow prior beliefs and attend the task based on the believability rather than the logic or any rules required of the task [35,37].

Unlike trained philosophers, it is difficult for most lay participants to suppress prior beliefs [29] and reason with the given premises in moral dilemmas despite being told that the situation is hypothetical. Greene and colleagues [11] also found that participants were unable to suspend their ‘real-world beliefs’ concerning the likelihood of the consequences of their decision in a moral dilemma. The authors tentatively concluded that ‘unconscious realism’ might be an important factor in hypothetical moral dilemmas. The current findings affirm these suspicions, and the conclusions drawn from our work–that prior beliefs and experiences may be influencing participants’ evaluation of the expected utility and morality of an action–indicate the pervasiveness of this inability to suspend belief and the potential consequences of such a discord between experimenter and participants.

While the current studies reinforced the hypothesis that the perceived uncertainty in moral dilemmas can explain a substantial amount of variance in the endorsement of sacrifice in moral dilemmas, it did not fully account for the discrepancy between impersonal and personal dilemmas. To complement the findings in this paper, we calculated participants’ subjective expected value (the number of lives that can be saved) of K and *~K* given the probability judgments provided by participants. We then examined the proportion of subjects who chose the option with higher subjective expected value. Results are presented in S1 Table, and indicate that not all participants base their moral judgments on the principle that their choice has the best expected outcome. Similar findings were also reported in Shou and Song [10], where participants’ perceived outcome probabilities did not fully align with their choices.

In addition, when the outcome probabilities were constrained to be exactly 100% in Study 2 and the expected outcome value of *K* was explicitly defined as higher than the expected outcome value of *~K*, not all participants chose *K*. These findings imply that the expected outcome value alone is an insufficient explanation for choice preference among participants in all moral dilemmas. As suggested by Baron and Spranca [38], ‘protected values’, which are values that resist trade-off for other values (e.g., economic) between two choices, can vary across different moral dilemmas. This implies that, although consequentialist reasoning has been shown to influence moral choices, deontic reasons also seem to play a role. Participants may perceive a higher cost/protected value in some dilemmas, such as the footbridge case, than other dilemmas, such as the switch case, and this consideration may be altogether independent of reasoning based on the outcomes of the moral dilemma.

Another possible explanation for the finding in Study 2 is that prior belief and belief bias could distort participants’ interpretation of ‘100%’. A statement with ‘100%’ can be less deterministic if participants held doubt about the statement in the first place. This may explain why in the numeric condition, although it significantly increased participants’ preference of killing, the increase was limited.

Furthermore, although the current study indicated that people do not interpret ‘will’ in the same way as 100%, some may argue that the use of the numeric information may promote or prime people to think more analytically [39,40]. Priming people to use deliberate processing can increase the endorsement of instrumental harm, which is a critical dimension of utilitarianism that most sacrificial moral dilemmas rely on [41]. The greater tendency to have utilitarian choices can be influenced by the increasing endorsement of utilitarian reasoning. However, a recent study showed that inducing more intuitive thinking by putting individuals under time pressure and cognitive load did not significantly increase utilitarian moral judgments [42]. Nevertheless, it would be interesting to investigate what probabilities they are actually assigning to the ‘will’ statement. Shou and Song [10] manipulated the probability information in different choice statements and found that altering the probabilities (e.g., changing the probability from 100% to 80%, 50% and 20%) significantly influenced participants’ endorsement of utilitarian options. Approximately 80% of participants eventually alter their decisions with the change of the probability information. A comparison between a lower probability (<100%) condition with the ‘will’ condition can be informative in terms of understanding how participants assign probability to the ‘will’ statement.

In terms of the role of emotions in judgment and decision making, as described by the dual-process theory, we do not deny the impact of emotion on moral judgments. While the dual-process theory implies that emotion can result in a change in the reasoning approach (e.g., from consequentialist reasoning to deontological reasoning), we suspect that emotion may influence moral judgments by biasing probability estimates and value judgments [16]. In Shou and Song [43], it was found that when the probability of one person dying in *K* (*P*(Loss|*K*)) was fixed at 100%, most subjects who initially chose *K* shifted their decision as soon as the probability that five people would die in *~K* was lower than 100. This also means that subjects who perceived that a 100% chance that one person dies (expected loss is one person) was morally worse than a 50% chance that five people die (expected loss is 2.5 people). This is possibly due to uncertainty aversion or intolerance, which is well documented in the decision-making literature [8]. People prefer a gamble with an uncertain loss (e.g., the chance that 5 people will die is lower than 100%) over a gamble with a certain loss (the chance that the individual dies is 100%). Nevertheless, future research should look at how effects of emotion and probability estimates interact in moral judgments.

### Implications and future directions

The present research reinforces our argument that perceived outcome probabilities vary across dilemmas. This also means that the subjective expected value of the outcomes for different actions vary across dilemmas. The variation in beliefs of outcome likelihoods is partially due to the inevitable limitations imposed by the descriptive nature of the tasks and the use of hypothetical scenarios. Researchers studying moral dilemmas have mistakenly assumed that participants interpret scenario outcomes as certain. Because participants perceive these outcomes as uncertain, perceived uncertainty influences their choices. Researchers should be encouraged to consider practices and investigations that better control such effects. For example, participant ratings of uncertainty regarding dilemma outcomes can be obtained and considered as covariates to account for the influence of perceived uncertainty on participants’ judgements and choices (as suggested by [10]). In addition, researchers could investigate ways of experimentally controlling or manipulating perceived outcome uncertainty so as to better understand its effects. However, we acknowledge that eliciting and controlling for probability judgments deals with only one among the many difficulties in designing scenarios that are truly comparable. Any two commonly used dilemmas may differ on multiple dimensions in addition to the intended dimension of interest.

The current study also highlights the potential influence of lay participants’ real-life judgment. Future studies of moral dilemmas should consider the ecological validity of descriptive tasks and move beyond. For example, there has been an increased popularity in endorsing virtual reality technology in moral judgment studies [44–46], and several studies have dependably replicated previous laboratory findings. In addition, there has been some discussion on the discrepancy between people’s responses in the hypothetical moral situations and their actual real life moral behaviors [47,48]. Of course the real life behaviors are constrained by a number of factors other than one’s moral values, such as social and interpersonal pressure. Future studies should consider how other factors may interact in influencing people’s moral judgments.

In sum, the findings of the current paper have far-reaching ramifications for the experimental literature concerned with moral dilemmas. Our findings illustrate a discord between experimenter and participant in the interpretation of task instructions. The descriptive data concerning the preferred behavior of moral agents in the countless experimental studies on trolley dilemmas are necessarily inexact due to the unaccounted for influence of uncertainty that pervades such judgments. The area of moral psychology concerned with moral dilemmas, as well as the broader field of judgment and decision-making, would benefit immensely if future research were directed at teasing out the moderating factors that engender such a discrepancy in the interpretation of task instructions.

## Supporting information

S1 Text Dilemmas used in the present study. (PDF)

S1 Table Proportions of choices aligning with subjective expected utility across dilemmas. (PDF)
