# Supplementary material for: Impact of uncertainty and ambiguous outcome phrasing on moral decision-making
Source: PLoS One. 2020 May 26;15(5):e0233127. doi: 10.1371/journal.pone.0233127 (PMC7250437; doi:10.1371/journal.pone.0233127)
Supplement: S1 Text — (PDF) [file pone.0233127.s001.pdf]

S1 Supplementary Text

Dilemmas used in the present study

Footbridge (Personal; retrieved from Greene et al., 2008):
In the path of a runaway train car are five railway workmen who will surely be killed unless you, a bystander, do something. You are standing on a pedestrian walkway that arches over the tracks next to a large stranger. Your body would be too light to stop the train, but if you push the stranger onto the tracks, killing him, his large body will stop the train. *Which do you think is morally better:*

Decision alternatives:
• Push the stranger onto the tracks to save the five lives
• Do not push the stranger

Probability judgment questions:
• If you choose to push the stranger onto the tracks, how likely do you think it is that the five workers on the tracks will survive?
• If you choose to push the stranger onto the tracks, how likely do you think it is that the stranger will die?
• If you choose to do nothing, how likely do you think it is that the stranger standing on the overpass will survive?
• If you choose to do nothing, how likely do you think it is that the five workers on the tracks will die?

Submarine (Personal; retrieved from Greene et al., 2008):
You are the captain of a small military submarine traveling underneath a large iceberg. An on-board explosion has dramatically decreased oxygen supply and has injured a member of the crew. He may die from his injuries. The remaining oxygen is not sufficient for the entire six-man crew to make it to the surface. If you shoot the injured crew-member, there will be just enough oxygen for everyone else to survive. *Which do you think is morally better:*

Decision alternatives:
• Shoot the crew member to conserve oxygen for the rest of the crew
• Do nothing

Probability judgment questions:
• If you choose to shoot the injured crewmember, how likely do you think it is that the rest of the crew will survive?
• If you choose to shoot the injured crewmember, how likely do you think it is that the injured crewmember will die?
• If you choose not to shoot the injured crewmember, how likely do you think it is that the injured crewmember will survive?
• If you choose not to shoot the injured crewmember, how likely do you think it is that the rest of the crew will die?

Safari (Personal; retrieved from Greene et al., 2008):
You are traveling with five other peace advocates in a war-torn country when your group is taken hostage by terrorists. After a week, the terrorists' demands have not been met, and the

leader offers you a choice: either they will kill all of you, or, if you shoot one of your fellow hostages, killing him, you and the others are free to go. *Which do you think is morally better:*

Decision alternatives:
• Shoot your fellow hostage so that you and the others are free to go
• Do not shoot your fellow hostage

Probability judgment questions:
• If you choose to shoot your fellow hostage, how likely do you think it is that the rest of the peace advocates will survive?
• If you choose to shoot your fellow hostage, how likely do you think it is that the this hostage will die?
• If you choose not to shoot your fellow hostage, how likely do you think it is that this hostage will survive?
• If you choose not to shoot your fellow hostage, how likely do you think it is that the rest of the peace advocates will die?

Transplant (Personal; retrieved from Greene et al., 2008):
You are a surgeon with a number of patients. Five of them need organ transplants. Each of them needs a different organ or they will surely die. You have another patient who is healthy and would be an ideal organ donor for the others. If you transplant his organs into the bodies of the other patients, they will live but he will die. *Which do you think is morally better:*

Decision alternatives:
• Perform the organ transplant to donate to the five others
• Do not perform the organ transplant

Probability judgment questions:
• If you choose to perform the transplant, how likely do you think it is that the five recipients will survive?
• If you choose to perform the transplant, how likely do you think it is that the organ donor will die?
• If you choose not to perform the transplant, how likely do you think it is that the organ donor will survive?
• If you choose not to perform the transplant, how likely do you think it is that the five potential recipients will die?

Sacrifice (Personal; retrieved from Greene et al., 2008):
You, your spouse, and your four children are crossing a mountain range on your return journey through the Highlands. You have inadvertently set up camp on a local clan's sacred burial ground. The leader of the clan says that according to the local laws, you and your family must be put to death. However, he will let yourself, your spouse, and three of your children live if you kill your oldest son. *Which do you think is morally better:*

Decision alternatives:
• Comply with the tribal leader's request
• Do not comply with the tribal leader's request

Probability judgment questions:

• If you choose not to obey the tribal leader's demand, how likely do you think it is that you and the rest of your family will survive?
• If you choose to obey the tribal leader's demand, how likely do you think it is that your son will die?
• If you choose not to obey the tribal leader's demand, how likely do you think it is that your son will survive?
• If you choose not to obey the tribal leader's demand, how likely do you think it is that you and your family will die?

Lifeboat (Personal; Greene et al., 2008):
You are on a small ship, a fire breaks out, and the ship has to be abandoned. Because your tiny life raft is carrying more than its capacity, it is sitting dangerously low in the water. The seas get rough and the raft begins to fill with water. Unless you do something, all six of you on the life raft will drown. There is an injured person on-board who may die either way; if you throw him overboard, everyone else will be saved. *Which do you think is morally better:*

Decision alternatives:
• Throw the injured person overboard to stop the boat from sinking
• Leave the injured person on the life raft

Probability judgment questions:
• If you choose to throw the injured person overboard, how likely do you think it is that everyone else on the life raft will survive?
• If you choose to throw the injured person overboard, how likely do you think it is that this person die?
• If you choose not to throw the injured person overboard, how likely do you think it is that the injured person will survive?
• If you choose not to throw the injured person overboard, how likely do you think it is that everyone on the life raft die?

Crying baby (Personal; retrieved from Greene et al., 2008):
Enemy soldiers have taken over your village and will kill all remaining civilians. You and five others are hiding in the cellar of a large house. Soldiers have come to search the house for valuables. A baby in your group begins to cry loudly. You cover the baby's mouth to block the sound, but it cannot breathe. If you remove your hand, the baby can breathe, but her crying will summon the soldiers who will kill everyone in the cellar. *Which do you think is morally better:*

Decision alternatives:
• Continue blocking the sound to prevent the soldiers from hearing
• Do not block the sound

Probability judgment questions:
• If you choose to continue blocking the sound, how likely do you think it is that everyone in the cellar will survive?
• If you choose to continue blocking the sound, how likely do you think it is that the baby will die?
• If you choose not to block the sound, how likely do you think it is that the baby will survive?

• If you choose not to block the sound, how likely do you think it is that soldier will find and kill everyone in the cellar?

Switch (Impersonal; retrieved from Greene et al., 2008):
In the path of a runaway train are five railway workmen who will surely be killed unless you, a bystander, do something. You see a nearby switch that, if you flip, will divert the train onto another track where it will kill a single railway workman. *Which do you think is morally better:*

Decision alternatives:
• Flip the switch to divert the train
• Do not flip the switch

Probability judgment questions:
• If you choose to flip the switch, how likely do you think it is that the five workers will survive?
• If you choose to flip the switch, how likely do you think it is that the one worker will die?
• If you choose not to flip the switch, how likely do you think it is that the one worker will survive?
• If you choose not to flip the switch, how likely do you think it is that the five workers will die?

Planecrash (Personal; retrieved from Greene et al., 2008):
Your plane has crashed in the Himalayas. The only survivors are you, four men, and a young boy. The six of you travel for days, battling extreme cold and wind. Your only chance of survival is to make it to a village a few days away. The boy is injured and cannot move very quickly. Without food, you and the other men will surely die. One of the men suggests suffocating the boy in his sleep and eating his remains over the next few days. *Which do you think is morally better:*

Decision alternative:
• Sacrifice the injured boy to save the rest of the survivors
• Do not sacrifice the boy

Probability judgment questions:
• If you choose to sacrifice the injured boy, how likely do you think that the group will survive?
• If you choose to sacrifice the injured boy, how likely do you think that the boy will die?
• If you choose not to sacrifice the injured boy, how likely do you think that the boy will survive?
• If you choose not to sacrifice the injured boy, how likely do you think that the group will die?

Fumes (Impersonal; retrieved from Greene et al., 2008):
You are the late-night watchman in a hospital where an accident has occurred in one of the on-site testing labs, and now there are deadly fumes rising up through the hospital's ventilation system. The fumes are headed to a certain area where there are five patients who will surely die. If you flip a switch, the ventilation system will cause the fumes to bypass this

room and enter a room containing a single patient, killing him. *Which do you think is morally better:*

Decision alternative:
• Divert the fumes into the single patients room so that they avoid the area with five people
• Do nothing

Probability judgment questions:
• If you choose to divert the fumes, how likely do you think it is that the five people in the other area will survive?
• If you choose divert the fumes, how likely do you think it is that the individual will die because of the fumes?
• If you choose to do nothing, how likely do you think it is that the individual will survive?
If you choose to do nothing, how likely do you think it is that the five people in the other area will die because of the fumes?

Burning (Personal; retrieved from Christensen et al. 2014):
You and five other people are trapped in a burning building. There is only one emergency exit through which all of you could escape, but it is blocked by burning debris. Another injured person is about to crawl through a hole at the bottom of the exit door. You and the four people behind you do not have time to do the same. If you use the injured person to break down the debris you will be able to escape. Doing this will kill him. *Which do you think is morally better:*

Decision alternatives:
• Break down the blockage using the injured person to save the group
• Continue waiting

Probability judgment questions:
• If you choose to break the blockage with the injured person, how likely do you think it is that the rest of the will survive?
• If you choose to break the blockage with the injured person, how likely do you think it is that the injured person dies?
• If you choose not to break the blockage, how likely do you think it is that the injured person will survive?
• If you choose not to break the blockage, how likely is it that the group behind will die?

Floods (Impersonal; retrieved from Christensen et al., 2014):
You are alone in a rescue helicopter during a devastating flood. You have rescued four critically injured people and you are keeping them alive. Suddenly, the pilot notifies you of a problem in the engine and gives the order that the helicopter needs to release weight or it will crash and you will all die. If you cut the cable by which the last rescued person is ascending, you will release enough weight to keep the helicopter in the air. This will kill him, but it will save you and the four people on board. *Which do you think is morally better:*

Decision alternatives:
• Cut the cord with the last survivor to keep the helicopter in the air
• Continue with all five survivors

Probability judgment questions:
• If you choose to cut the cable, how likely do you think it is that you and the four survivors will survive?
• If you choose to cut the cable how likely do you think it is that the last survivor ascending the cable will die?
• If you choose not to cut the cable, how likely do you think it is that the last survivor ascending the cable will survive?
• If you choose not to cut the cable, how likely do you think it is that the helicopter will crash?

Shipyard (Impersonal; retrieved from Christensen et al., 2014):
You are part of a shipyard dock team that attaches crane cables to containers to unload the cargo ships. You have just attached cables to a container and are now climbing on top of it to make sure it is unloaded properly. Suddenly the red warning light flashes indicating that a cable is about to fail. If it fails over the deck of the ship, the container will collapse onto five people below. If you push the emergency release button, the container will be dropped back into the cargo bay. You will be held suspended in mid air by your safety harness, but one crew member is still working in the cargo bay. *Which do you think is morally better:*

Decision alternatives:
• Push the emergency button to drop the container back into the cargo bay
• Allow the container to fall onto the deck

Probability judgment questions:
• If you choose to push the emergency button, how likely do you think it is that the workers on the deck below will survive?
• If you choose to push the emergency button, how likely do you think it is that the worker in the cargo bay will die?
• If you choose not to push the emergency button, how likely do you think it is that the worker in the cargo bay will survive?
• If you choose not to push the emergency button, how likely do you think it is that the workers on the deck below the container will die?

Sharks (Impersonal; retrieved from Christensen et al., 2014):
You and five divers are part of an U.N. team who is deactivating anti-ship mines from World War II. One team member has hurt himself and the blood in the water has attracted several sharks. The bleeding diver is swimming towards the last protective cage and will reach it before you and the four others.
If you detonate one of the mines by means of a remote control, it will kill the injured diver and the sharks will stop to eat him, allowing you and the other four to reach to cage. *Which do you think is morally better:*

Decision alternatives:
• Detonate the mine
• Do not detonate the mine

Probability judgment questions:

• If you choose to detonate the mine, how likely do you think it is that the group will reach the protective cage and survive?
• If you choose to detonate the mine, how likely do you think it is that the injured swimmer will die?
• If you choose not to detonate the mine, how likely do you think it is that the injured swimmer will survive?
• If you choose not to detonate the mine, how likely do you think it is that the group will die?

Scaffolding (Impersonal; retrieved from Christensen et al., 2014):
You and your construction crew are standing on some scaffolding, working on a skyscraper. Suddenly, the scaffolding collapses partially. You and five others are hanging on to a dangling crossbar, but it cannot hold the weight of all of you. The worker next to you slips off the crossbar and catches himself on another portion of the scaffolding. If you pull out a latch that will detach the section of scaffolding with your co-worker on it, this will remove just enough weight that the rest of you can make it to safety before the scaffolding collapses entirely. This will kill him but save you and the other five co-workers. *Which do you think is morally better:*

Decision alternatives:
• Pull out the latch and detach the section of scaffolding
• Do not pull out the latch

Probability judgment questions:
• If you choose to detach the scaffolding, how likely do you think it is that the you and the rest of the workers will survive?
• If you choose to detach the scaffolding, how likely do you think it is that the worker on the other portion of scaffolding will die?
• If you choose not to detach the scaffolding, how likely do you think it is that the worker on the other portion of scaffolding will survive?
• If you choose not to detach the scaffolding, how likely do you think it is that you and the crew of workers die?

Bikers (Impersonal; retrieved from Christensen et al., 2014):
You are an expert motorcyclist participating in a Bike Week. As you are driving down the road in front of a group of five bikers, you suddenly notice that a biker up front is losing control over his machine and is falling. You can easily evade him yourself but the bikers behind you will crash into each other and die in the resulting pile-up. If you let your bike run into the falling biker while jumping into the roadside ditch yourself, you will warn the others in time. This will kill this biker, but you will save the other five bikers behind you. Which do you think is morally better:

Decision alternatives:
• Ditch your motorcycle into the rider in front of you, warning the bikers behind you
• Evade the bike in front

Probability judgment questions:
• If you choose to ditch your bike into the rider in front of you, how likely do you think it is that the five bikers behind you will survive?

• If you choose ditch your bike into the rider in front of you, how likely do you think it is that the biker in front of you will die?
• If you choose to evade the biker in front of you, how likely do you think it is that the biker in front of you will survive?
• If you choose to evade the biker in front of you, how likely do you think it is that the five bikers behind you will die?

Car (Impersonal; retrieved from Shou & Song, 2016):
You are driving a truck and will reach a hairpin turn near a cliff soon. A car in front of you suddenly stops at the edge of the cliff. You noticed earlier that there are five people inside the car. Even if you brake, it is too late for you to fully stop the truck as the truck is heavily loaded. Your immediate response to avoid hitting that car is to turn your truck left. However, you notice that if you turn left, you are going to hit a passer-by who is walking on the left side of the road. *Which do you think is morally better:*

Decision alternatives:
• Immediately turn left, hitting the passer-by but avoiding the car in front of you
• Allow the truck to hit the car in front of you

Probability judgment questions:
• If you choose to turn left, how likely do you think it is that the group of people in the car in front of you will survive?
• If you choose to turn left, how likely do you think it is that the passer-by to your left will die?
• If you choose to continue going straight, how likely do you think it is that the passer-by to your left will survive?
• If you choose to continue going straight, how likely do you think it is that the group in the car in front of you will die?

Miners (Impersonal; retrieved from Christensen et al., 2014):
You are a miner. The only way out of the mine is to ride up with a simple cable elevator with small cubicles. While you are in the queue, you see that the cable supporting all the cubicles is about to snap. Six miners are riding the cubicles at this time. If you push an emergency switch the last cubicle with only one miner inside will be detached from the cable. This will reduce enough weight to maintain the cubicles of the other five miners. This will kill the miner in the detached cubicle, but it will save the other five. *Which do you think is morally better:*

Decision alternatives:
• Detach the last cubicle
• Do not detach the last cubicle

Probability judgment questions:
• If you choose to detach the last cubicle, how likely do you think it is that the workers in the other cubicles will survive?
• If you choose to detach the last cubicle, how likely do you think it is that the lone worker in the last cubicle will die?
• If you choose not to detach the last cubicle, how likely do you think it is that the lone worker in the last cubicle will survive?

• If you choose not to detach the last cubicle, how likely do you think it is that the workers in the rest of the cubicles die?

Example Questions from the ‘Preferred’ and ‘Not Preferred’ Conditions of the Post-hoc Justification Check

Not preferred condition decision alternatives for the footbridge dilemma:
• Push the stranger in front of the tracks to stop the train
• Do nothing, allowing the train to continue on the track it is currently on
• Pull a nearby lever that would derail the train, killing most on board

Preferred condition decision alternatives for the footbridge dilemma:
• Do nothing, allowing the train to continue on the track it is currently on
• Push the stranger in front of the tracks to stop the train
• Divert the train onto a new track with no one on it via a nearby switch
