# Supplementary material for: Impact of uncertainty and ambiguous outcome phrasing on moral decision-making
Source: PLoS One. 2020 May 26;15(5):e0233127. doi: 10.1371/journal.pone.0233127 (PMC7250437; doi:10.1371/journal.pone.0233127)
Supplement: S1 Table — (PDF) [file pone.0233127.s002.pdf]

S2 Table. Proportions of choices aligning with subjective expected utility across dilemmas

| Impersonal Dilemma | Proportion (%) | Personal Dilemma | Proportion (%) |
|---|---|---|---|
| Switch | 0.54 | Burning | 0.32 |
| Fumes | 0.54 | Lifeboat | 0.52 |
| Shipyard | 0.83 | Submarine | 0.39 |
| Car | 0.61 | Plane crash | 0.25 |
| Floods | 0.56 | Transplant | 0.24 |
| Miners | 0.57 | Footbridge | 0.24 |
| Scaffolding | 0.50 | Crying baby | 0.62 |
| Bikers | 0.51 | Sacrifice | 0.25 |
| Sharks | 0.39 | Safari | 0.27 |
